# Clinical Heterogeneity, Unmet Needs and Long-Term Outcomes in Patients with Systemic Lupus Erythematosus

**DOI:** 10.3390/jcm11226869

**Published:** 2022-11-21

**Authors:** Christopher Sjöwall, Ioannis Parodis

**Affiliations:** 1 Department of Biomedical and Clinical Sciences, Division of Inflammation and Infection/Rheumatology, Linköping University, SE-581 85 Linköping, Sweden; 2 Department of Medicine Solna, Division of Rheumatology, Karolinska Institutet and Karolinska University Hospital, SE-171 76 Stockholm, Sweden; 3 Department of Rheumatology, Faculty of Medicine and Health, Örebro University, SE-701 82 Örebro, Sweden

## 1. Introduction

The clinical presentation of systemic lupus erythematosus (SLE) is highly heterogeneous, ranging from mild disease limited to skin and joint involvement to life-threatening conditions with renal impairment, severe cytopenias, central nervous system disease, and thromboembolic events [1]. Despite significant advances in our understanding of the pathophysiology and optimization of medical care, SLE populations still exhibit premature mortality. Many patients with SLE experience poor health-related quality of life (HRQoL), even after successful treatment in terms of clinical and laboratory parameters [2], as well as severe disease flares with an increased risk of organ damage [3]. The development of effective drugs for SLE—which can induce remission or lower disease activity—the unanimous use of definitions of remission and low or high disease activity, flare, and response to therapy, the identification of non-invasive biomarkers of disease activity and long-term outcomes, and the implementation of the patient perspective as an integral part of the clinical assessment constitute only a few of the many unmet needs in the field of SLE.

In this Special Issue hosted by the Journal of Clinical Medicine, we selected a series of articles that highlight current and contribute new knowledge related to aspects such as clinical heterogeneity, autoantibodies, and long-term outcomes in SLE. Several of the contributions focus on patients’ perspectives and SLE patients’ unmet needs, for instance, fatigue, poor HRQoL experience, and non-adherence to medications. In this Editorial, we provide an overview of challenges and opportunities in the management of SLE, as presented by authors who contributed to the collection, and we hope that this will prove valuable both for clinicians and people living with SLE.

## 2. Clinical Heterogeneity

In a study by Jung and co-authors, hierarchical clustering was performed to gain insights into the clinical heterogeneity of SLE [4]. Three distinct clusters of patients with different manifestations and antibody profiles were identified among 389 patients through the combination of laboratory test results at SLE onset and linear discriminant analysis, utilized to construct prediction models. In a comprehensive review, Mahler et al. summarized the history and future directions regarding anti-Ki/SL antibodies in SLE and Sjögren’s syndrome, which were first described in 1981 [5].

Register data can be used to evaluate changes over time. Moreno-Torres et al. used the Spanish national registry to evaluate trends in hospital admissions and causes of death from the late 1990s until 2015 using ICD codes [6]. The authors concluded that the improved control of SLE over the past two decades has led to a decrease in early admissions to hospital and disease chronification. In line with data from other groups [7,8], cardiovascular disease, infections, malignancies, and thromboembolic events were among the most common causes of death.

Similarly, ICD codes and reliable national patient register data were used during the coronavirus disease 2019 (COVID-19) pandemic to improve our understanding and patient care. In this Special Issue, Cordtz and colleagues demonstrated that Danish patients with SLE were at an approximately threefold increased risk of hospitalization due to COVID-19 compared with age- and sex-matched comparators from the general population [9]. Interestingly, no obvious impact of the use of glucocorticoids or hydroxychloroquine was seen on the risk of hospitalization.

Diagnosis of autoimmune liver diseases (AILD) among individuals with an already established diagnosis of SLE is challenging since liver enzyme test abnormalities and hypergammaglobulinemia are common laboratory findings in SLE, and antinuclear antibodies (ANA) constitute a prerequisite. Heijke et al. demonstrated why the autoimmune hepatitis criteria [10] are less useful in SLE populations and why a liver biopsy should be performed with the measurement of other AILD-associated autoantibodies [11].

## 3. Patient-Reported Experience

Several contributions in this Special Issue dealt with patient-reported outcome measures (PROMs), mainly PROMs capturing SLE patients’ HRQoL.

Nguyen and colleagues reviewed the literature for the use of PROMs to assess HRQoL, both in research settings and clinical practice, and described the characteristics of commonly used PROMs [12]. The authors advocate that the increased use of PROMs may help alleviate the discordance of health perception between patients and clinicians, which would be especially useful for patient populations with a high comorbidity burden.

Lai et al. compared the well-established SLE disease activity index 2000 (SLEDAI-2K) with the more recent SLE disease activity score (SLE-DAS) in terms of their correlation with the Lupus Quality of Life questionnaire (LupusQoL) in Taiwanese SLE patients and found that both activity indices perform fairly well in capturing SLE patients’ health experience, with no substantial differences [13].

Fatigue is a common and multifaceted phenomenon in SLE, oftentimes neglected by clinicians. One of the reasons for this is due to the scarceness of interventions that have shown effectiveness in improving fatigue. Kawka and colleagues reviewed the literature to shed light on the impact, determinants, and management of fatigue in patients with SLE [14]. Some pharmaceuticals have demonstrated ability to alleviate fatigue, as has non-pharmacological management such as psychosocial interventions and lifestyle improvements. The authors suggest that the management of fatigue in SLE should rely on person-centered approaches and targeted interventions. Fatigue was also addressed in a review by Dey et al., which demonstrated how fatigue is manifested and managed in patients with SLE and rheumatoid arthritis (RA) [15]. While the two diseases differ in terms of clinical manifestations, fatigue is commonly reported in both patient populations. It is associated with pain, depression, and anxiety, and affects function, work capacity, and quality of life. Comorbidities contribute to fatigue, further complicating its management. Collectively, fatigue should be managed in a holistic manner, along with the management of comorbidities and management of factors that augment its impact on patients’ lives.

As a tool to fight fatigue, a study from Sweden by Skoglund and colleagues revived an old mechanism that has shown promise, namely the adrenal hormone dehydroepiandrosterone (DHEA) [16]. The authors studied DHEA dosages in relation to SLE activity, glucocorticoid use, concomitant immunosuppressants, and patient-reported pain, fatigue, well-being, HRQoL, and functional disability. DHEA treatment was safe but did not alter disease activity or organ damage progression over time. Some improvement was seen regarding fatigue; however, this did not reach statistical significance. The authors nevertheless suggested that the determination of DHEA blood concentrations should be performed prior to treatment commencement, along with the exclusion of comorbidities that may require other therapeutic approaches.

Undoubtedly, one of the major challenges in SLE management is posed by non-adherence to therapy. Emamikia and colleagues interviewed patients with SLE from two Swedish centers in a qualitative study aiming at identifying influenceable contributors to non-adherence and suggesting interventions to alleviate this phenomenon [17]. The reasons for non-adherence were complex and multifaceted, both intentional and unintentional, related to the relationship between patients and caregivers, lack of information about the disease and medications, and influence from family and friends. Increased communication between patients and caregivers, patient education, psychosocial support, and the involvement of family members in the patients’ journey through their disease were some of the potential contributors that the authors suggested for the increased adherence of SLE patients to their medications.

Anxiety and depression are major concerns in patients with SLE. Nikoloudaki et al. examined longitudinal trends in anxiety, depression, and SLE activity and showed that a high mental disease burden persists despite disease control in some patients [18]. Based on these findings, the authors suggested that socioeconomic facets should be a part of comprehensive patient evaluations. Importantly, and to make the connection with the study by Emamikia et al. [17], anxiety and depression were associated with non-adherence to medications.

## 4. Long-Term Outcomes

Longitudinal follow-up of patients with SLE using validated tools to assess disease activity and organ damage is crucial for understanding the true nature and burden of the disease. Gerosa et al. reported data from the Milan SLE consortium cohort (SMiLE) with an impressively long follow-up and data on the attainment of remission and lupus low-disease-activity state (LLDAS) [19]. In line with observations of other groups [20], the authors demonstrated that the attainment of remission or LLDAS was associated with less organ damage. Furthermore, this study showed that patients with a longer distance from disease onset are at a higher risk of developing disease flares, which in turn constitutes a risk factor for late damage accrual.

The potential genetic background underlying damage development in distinct organ domains was the focus of a study by Ceccarelli and colleagues [21]. The authors provided new insights into the genetic susceptibility for damage accrual in the renal and neuropsychiatric domains in particular, based on gene polymorphisms.

In a systematic literature review, Reppe Moe et al. compiled data on mortality, end-stage kidney disease (ESKD), and cancer from population-based studies, and found that cardiovascular disease was the most frequent cause of death over 15 years of follow-up. Moreover, 5–11% of patients developed ESKD, and no evidence for increased cancer incidence was found [22].

Last but not least, Suzon et al. summarized long-term population-based data in Afro-descendant patients with lupus nephritis (LN) from Martinique [23]. The main purpose of this study was to determine the rates of ESKD and mortality in an Afro-descendant LN population with a generally high income as well as easy and free access to healthcare. Unlike the stale notion that patients of African descent overall have worse ESKD and mortality rates compared to Caucasians, this study reported overall favorable rates, comparable to those seen in Caucasians, estimated at 21.3% for ESKD and 7.9% for mortality at 20 years of follow-up. These results underscore the importance of optimizing modifiable contributors to poor outcomes, especially socioeconomic factors.

## 5. Perspective

Herein, we present a rich collection of important contributions by esteemed colleagues in the field of SLE and autoimmunity, ranging from disease classification and genetic susceptibility to disease evolution to facilitators towards improved long-term outcomes and patient-reported health experience. We firmly believe that this article collection contributes to novel knowledge and substantiates older, well-established notions, all presented in a manner that provides direct clinical implications and emphasizes the importance of incorporating the patient perspective in a holistic, patient-centered, and tailored management of people living with SLE.

The articles of this Special Issue will also be made available in the form of an electronic book. We would like to express our gratitude to all colleagues who contributed works to this Special Issue and look forward to seeing the findings reported herein being discussed and implemented in clinical practice, making an impact and ultimately a difference in SLE patients’ lives.
